# Supplementary material for: Content-rich biological network constructed by mining PubMed abstracts
Source: BMC Bioinformatics. 2004 Oct 8;5:147. doi: 10.1186/1471-2105-5-147 (PMC528731; doi:10.1186/1471-2105-5-147)
Supplement: Additional File 2 — The original results of the above study (non-essential files are deleted to keep the file size under the limit set by BMC bioinformatics). [file 1471-2105-5-147-S2.bz2 › chilibotAdditionalFile2/dip05/96ID9324938E403/html/BAD_BCL2L1.html]

 


 **BAD** and **BCL2L1** 
  
Found 251 abstracts in PubMed, retrieved 05.  
 

 What does Google say? 
 PDF only 
| .edu only 

---

**Interactive relationship** (e.g. stimulation, inhibition, etc)

**Stimulatory relationship**- In WtFLT3 transfected 32D WtFLT3 32D cells, FLT3 ligand FL stimulation did not restore the down regulation of Bcl XL  [ **BCL2L1** ]  but maintained the phosphorylation of  **Bad** .  Ref: 12842996 Blood< MedlineTA>Blood,
**Neutral relationship**- We describe the implementation of confocal and two photon excitation fluorescence resonance energy transfer FRET microscopy techniques through which we demonstrate interaction between the proapoptotic protein  **BAD**  and the prosurvival protein Bcl xL  [ **BCL2L1** ]  within TAI following TBI.  Ref: 12880338 J Biomed Opt, 2003

**Non-interactive relationship** (e.g. studied together, co-existance, homology, etc.)

- One proposed pathway by which this has been hypothesized to occur is the Ca 2 mediated activation of calmodulin and subsequent activation of the phosphatase calcineurin with dephosphorylation of a protein known as  **BAD** , leading to a proapoptotic interaction between  **BAD**  and the mitochondrial protein Bcl xL  [ **BCL2L1** ] .  Ref: 12880338 J Biomed Opt, 2003
- While this pathway is an intriguing route for traumatic axonal pathogenesis, neither conventional immunocytochemical histochemical nor ultrastructural approaches have had the capacity to shed insight on whether  **BAD**  and Bcl xL  [ **BCL2L1** ]  interact in TAI in vivo.  Ref: 12880338 J Biomed Opt, 2003
- Furthermore, the dephosphorylation of  **Bad**  using LY294002 and PD98059 was insufficient for apoptosis, and the down regulation of Bcl XL  [ **BCL2L1** ]  using antisense treatment was needed to induce apoptosis.  Ref: 12842996 Blood< MedlineTA>Blood, 2003
- In a murine myeloid cell line, 32D, IL 3 deprivation induced apoptosis following the down regulation of Bcl XL  [ **BCL2L1** ]  and the dephosphorylation of  **Bad** .  Ref: 12842996 Blood< MedlineTA>Blood, 2003
- FLT3 kinase inhibitor, AG1296, alone not only dephosphorylated  **Bad**  but also down regulated Bcl XL  [ **BCL2L1** ] , leading FLT3 ITD 32D cells into apoptosis.  Ref: 12842996 Blood< MedlineTA>Blood, 2003
- Furthermore, Bcl 2 family members  **Bad** , Bak, and Bcl xS  [ **BCL2L1** ]  protein levels were increased in FHIT transfected clones when compared with Panc 1 cells.  Ref: 12890991 Pancreatology, 2003
- Remarkably, these events occurred in the absence of any reduction in the expression of the Bcl 2 family members Bcl 2, Mcl 1 and Bcl XL  [ **BCL2L1** ] , or any change in the proapoptotic molecules  **Bad**  or Bax.  Ref: 12842827 Am J Physiol Gastrointest Liver Physiol< MedlineTA>Am J Physiol Gastrointest Liver Physiol, 2003
